# Case report: 11 years on hemodialysis with a 4-year-old baby girl: A success story

**DOI:** 10.3389/fmed.2022.1091568

**Published:** 2023-01-25

**Authors:** Alex Tatang Mambap, Efuetnkeng Bechem, Kate Mafor Kan, Sylvain Njoyo Laah, Frida Sunjoh, Gloria Enow Ashuntantang

**Affiliations:** ^1^Department of Clinical Sciences, Faculty of Health Sciences, The University of Bamenda, Bamenda, Cameroon; ^2^Bamenda Regional Hospital, Bamenda, Cameroon; ^3^Department of Internal Medicine and Specialties, Faculty of Medicine and Biomedical Sciences, The University of Yaoundé I, Yaoundé, Cameroon

**Keywords:** pregnancy, outcome, hemodialysis (HD), Cameroon (Sub-Saharan Africa), Bamenda

## Abstract

Despite advances in clinical management and dialysis care, the outcome of unplanned pregnancy in women on maintenance hemodialysis (MHD) remains a difficult journey for the patient, fetus, and healthcare staff, particularly in low-resource countries. We report the successful outcome of a pregnancy in an anuric woman on twice-weekly maintenance hemodialysis for chronic glomerulonephritis since November 2012 in Cameroon. She was discovered pregnant at 18 weeks of gestation. The pregnancy was maintained until 36 weeks when a healthy 2,270 g female baby was delivered by elective cesarean section for tight nuchal cords and intrauterine growth retardation. The mother’s post-partum period was uneventful. Except for hypoglycemia shortly after birth, the baby was fine. The patient is still on hemodialysis after 4 years, and the child is healthy and attending school.

## Background

Pregnancy on maintenance hemodialysis (MHD) is shifting from being an exception to being a rare, but not an impossible event (1). However, this remains exceptional in resource-limited settings where dialysis adequacy is unachievable due to inadequate funding, absence of health coverage, and limited access to both dialysis and corrective therapies for end-stage kidney disease (ESKD) (2). To date, the conception and successful outcome of these pregnancies are much more frequent when the patients have a residual kidney function, a shorter HD vintage, and adequate dialysis efficiency and pharmacologic treatment (1). However, when pregnancy occurs under such precarious conditions, it becomes a very challenging journey for the patient, the fetus, and the healthcare staff. Despite an increasing number of successful pregnancies in developed countries (1, 3–5), there are sparse reports of successful pregnancies in women undergoing MHD in Sub-Saharan Africa where dialysis is heavily self-funded (1, 2).

In Cameroon, Ashuntantang et al. reported in 2014 a conception rate of 7.14% over 11 years in a series of 84 women of childbearing age undergoing MHD (2). To date, only two cases of successful pregnancies in women on MHD have been reported in the country (2, 6). These two successful cases have been reported in first-category hospitals in patients on hemodialysis for less than 24 months with residual kidney function. We, hereby, report the first successful pregnancy in an anuric lady after 7 years on a twice-weekly MHD in a third-category hospital in Cameroon. The patient provided written consent for publication of the case.

## Case presentation

Ms. X was a 29-year-old woman in 2017, who had been on MHD since November 2010 for severe uremic encephalopathy secondary to end-stage kidney disease, presumed to be secondary to hepatitis B virus-related glomerulonephritis. She/the patient became anuric 3 years after the initiation of dialysis. Her dialysis routine consisted of two HD sessions of 4 h weekly (on Wednesday and Saturday; occasionally once a week during stock-outs of HD supplies), using bicarbonate dialysate and polysulfone dialyzer with a surface area of 1.8 m^2^. HD was performed using the Fresenius 4008S generator (Fresenius Medical Care Homburg, Germany). Her regular medications included amlodipine 10 mg/day and calcium carbonate. She occasionally received alfacalcidol 0.25 μg three times per week depending on the availability of funds. She received frequent blood transfusions for anemia correction and became hepatitis C positive after 3 years on HD. She was single, nulliparous, had never been pregnant, and had a very irregular menstrual cycle for the past 2 years.

**FIGURE 1:**
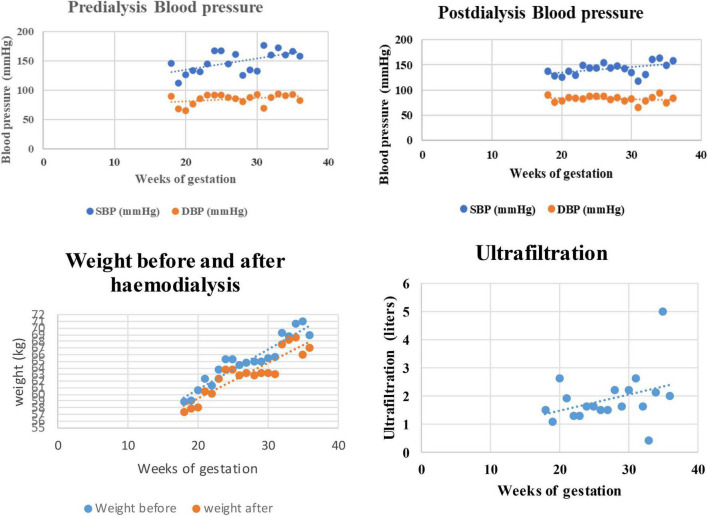
Blood pressure, weight, and ultrafiltration with the gestational age of the mother.

**FIGURE 2:**
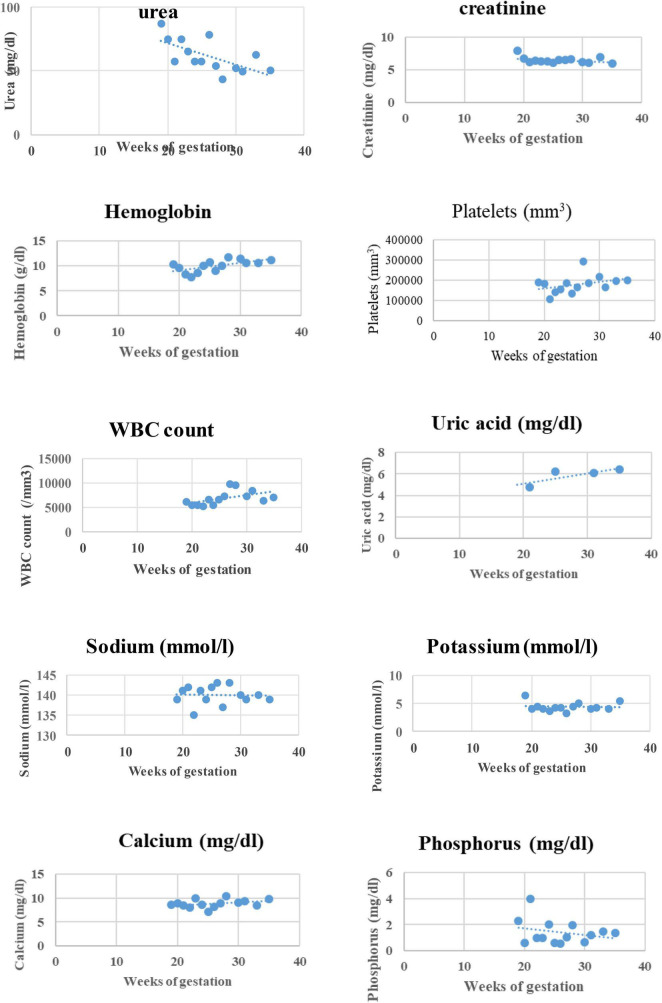
Evolution of weekly biochemical parameters with gestational age.

She consulted on 20 September 2017 for a distended and painless abdomen of 8 weeks duration. Abdominal distention had been progressive and was associated with an increase in food cravings and hunger pangs. Her ongoing medications were as usual. Further questioning and analysis of her HD record revealed an unusual increase in interdialytic weight gain of 2 kg and frequent intradialytic hypotension within the past 3 months before the consultation. On examination, blood pressure (BP) was 125/82 mmHg and weight was 56.75 kg. Abdominal examination revealed an increased uterus with a symphyseal-fundal height of 15 cm. Pelvic ultrasonography confirmed a live singleton intrauterine pregnancy estimated at 18 weeks of gestation with normal amniotic fluid volume. The biochemical work-up of the patient at the diagnosis of pregnancy is reported in Table 1. She/the patient was extensively counseled about the challenges of pregnancy on MHD; however, she opted to keep the pregnancy. According to previous reports on management guidance, changes were made in her dialysis care (5, 7). We then modified the HD regimen: the number of HD sessions was increased from 2 to 4 per week and the duration from 4 to 5 h each up to the 34th week of gestation, and then from 4 to 5 sessions of 5 h per week (25 h/week) till delivery. The surface area of the dialyzer was reduced to 1.5 m^2^ and the maximum blood flow rate and ultrafiltration rate were set at 250 ml/min and 400 ml/h, respectively. We introduced slow ultrafiltration to achieve a weight gain of 0.5 kg every fortnight and a target post-dialysis systolic blood pressure between 110 and 130 mmHg and diastolic between 80 and 90 mmHg (see Figure 1). Standard heparin was continued till 34 weeks of gestation after which it was substituted with enoxaparin to minimize the risk of hemorrhage. She was placed on 100 mg of aspirin daily and epoetin beta three times per week with the dose adjusted to maintain the maternal hemoglobin at 11–12 mg/dl. Other drugs introduced included: 100,000 UI of native vitamin D every 2 months, 500 mg of vitamin C twice per week, intravenous vitamin B complex at end of every HD session, 5 mg of folic acid daily, and 100 mg of intravenous iron sucrose weekly. Amlodipine was substituted for nicardipine and she continued calcium carbonate but between meals this time. She was also placed on a weekly dose of three tablets of a fixed combination of sulfadoxine 500 mg and pyrimethamine 25 mg for malaria prophylaxis as per standard local antenatal care practice. We did not limit the patient’s dietary intake except for salt restrictions, and the patient was encouraged to increase the intake of dairy products and animal protein to prevent hypophosphatemia in the absence of phosphorus tablets. Obstetric care consisted of 2 weekly standard care similar to non-HD pregnant women. Serial obstetric ultrasonography was performed monthly to assess fetal morphology and growth, placental blood flow, and amniotic fluid volume (Table 2). Pre-hemodialysis full blood count, serum creatinine, blood urea, sodium, potassium, phosphorous, and calcium were done weekly, while plasma uric acid and C-reactive protein were monitored monthly (see Figure 2). To attain these objectives, non-governmental organizations, individual sponsors, and the hospital provided material and financial assistance to cover all the costs of care. The evolution was uneventful, but a persistent increase in BP above the target at 28 weeks of gestation led to the addition of alpha-methyldopa (250 mg twice daily). Despite this, the BP continued rising without features of pre-eclampsia. Labetalol 400 mg/day was then added (Figure 1). Despite malaria prophylaxis, she had an episode of malaria at 25 weeks of gestation, which was successfully treated with quinine protocol.

**TABLE 1:** Initial biochemical work-up of mother.

Initial biological work-up	26/9/2017 (week 19)	Reference/norme
WBC (/mm^3^)	6,000	3,500–10,000
Hemoglobin (g/dl)	10.3	12–16
Platelets (/mm^3^)	189,000	150,000–440,000
CRP (mg/l)	20	<6
Predialysis urea (mg/dl)	87	15–45
Predialysis creatinine (mg/dl)	7.9	0.5–1.1
Sodium (mmol/l)	139	137–151
Potassium (mmol/l)	6.4	3.5–5.8
Calcium (mg/dl)	8.56	8.1–10.4
Phosphorus (mg/dl)	2.3	2.7–4.5
Uric acid (mg/dl)	4.8	2.5–6
AST	27.6	13–31
ALT	20.6	7–35
Prothrombin time (%)	98	70–100
HIV	Negative	Negative
AgHBs	Positive	Negative
AgHBe	Negative	Negative
AbHBc	Positive	Negative
HCV ab	Positive	Negative
VDRL	Negative	Negative
TPHA	Negative	Negative
Toxoplasmosis IG g (UI/L)	10	

**TABLE 2:** Ultrasound follow-up of the mother.

Variable	20/9/2017	19/10/2017	28/12/2017	19/1/2018
Estimation gestational age	18 wk+1 d	22 wk+3 d	30 wk+4 d	34 wk+1 d
Weight (grams)	222	485	1,541	2,340
Resistivity index	Normal	Normal	Normal	Normal+tight nuchal cords
Amniotic fluid	Normal	Normal	Normal	Normal
Foetal heartbeat	147	155	139	143
Morphology		Normal	Normal	Normal
Presentation			Cephalic	
Placenta			Grade 2 posterior	Grade 2 posterior

The patient was admitted at 36 weeks for elective cesarean section with the indication being tight nuchal cords and intrauterine growth retardation. On 24 January 2018, she underwent a cesarean section under spinal anesthesia with 10 mg of hyperbaric bupivacaine, and a 2,270 g female baby with an Apgar score of 9 at 1 min and 10 at 5 min was delivered 10 min after the beginning of surgery. The estimated blood loss was less than 200 ml. After the surgery, the mother was stable but for a blood pressure of 148/108 mmHg. She was then transferred to the intensive care unit (ICU). Her post-operative course was uneventful, and on the second day post-operatively, she resumed her pre-pregnancy hemodialysis schedule of 4-h sessions twice a week. She was discharged home on the 10th day post-operatively and is still on hemodialysis 4 years after. For the neonate, she was admitted to the neonatal care unit immediately after delivery where she presented with hypoglycemia of 60 mg/dl. No polyuria, no signs of dehydration, and no morphological malformations were observed. She was then placed on glucose 10%, calcium gluconate, and parenteral antibiotics. She also received hepatitis B immune globulins and a vaccine against hepatitis B to prevent maternal–fetal transmission. Due to the risk of maternal–fetal transmission of hepatitis B, the baby was exclusively fed with artificial milk. The baby evolved well and was discharged home on day 7.

**FIGURE 3:**
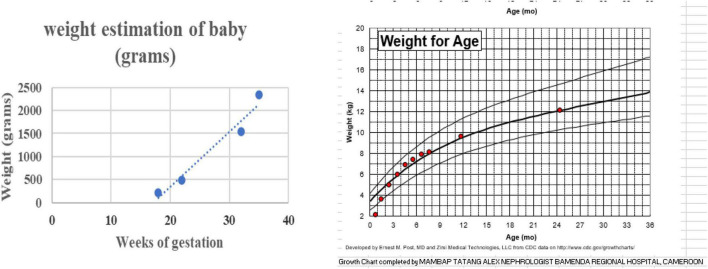
Intra-uterine and extra-uterine weight change of the child.

To this date, the child has never been hospitalized. She was fed on commercial breast milk substitutes from birth till 5 months. Gruel and cereals fortified with milk were introduced from 6 to 8 months. The semi-solid adult food was commenced from the ninth month, and she was fed 4–5 times a day with at least two milk drinks in her diet. Growth monitoring showed catch-up growth with the progressive acceleration of physical anthropometric parameters indicative of normal growth (see Figure 3). The developmental milestones acquisition was adequate for the age. She was followed up regularly by the pediatrician and reported five episodes of acute febrile illness with cough treated as an outpatient for acute uncomplicated respiratory tract infection. At 46 months, her blood pressure, renal ultrasonography, and laboratory results, including a full blood count, blood chemistry, serum creatinine, and urine dipstick, were essentially normal. AgHBs serology was negative (see Table 3).

**TABLE 3:** Summary of work-up of the child at 4 years.

Work-up	Results
Serum urea	11.5 mg/dl
Serum creatinine	0.6 mg/dl
Serum sodium	144 mmol/L
Serum potassium	5.3 mmol/L
Serum chloride	106 mmol/L
Serum calcium	10.5 mmol/L
Hepatitis B surface antigen	Negative
Urinalysis	Areactive SG: 1,015 pH: 6.5
Renal ultrasound	Both kidneys are normal sizes (The right measuring—6.5 cm in length and the left 6.9 cm with parenchymal thickness of 1.4 cm) with normal corticomedullary differentiation with normal calyces, ureter, and bladder

## Discussion

This is the third report of a successful pregnancy in a woman on MHD in Cameroon, however, this is the first case in an anuric woman after 7 years on MHD undergoing twice weekly hemodialysis sessions.

In Cameroon, the actual incidence of conception during ESKD is unknown but a cumulative incidence of conception among ESKD patients of childbearing age on MHD of 7.14% in 11 years was reported (2). In the early reports in Cameroon, conception occurred within the first year of hemodialysis (2, 6). This case report, however occurred after 7 years on MHD. The rate of conception and successful outcome of pregnancies on HD reduce with longer HD vintage of women (8). In addition, compared with previously reported cases in the country, our patient was anuric for many years. Most studies report more frequent conception and successful pregnancies when the patients have residual renal function (7, 8).

The diagnosis of pregnancy in our case was late (18 weeks of gestation) because her amenorrhea was not a new finding, and she had few suggestive symptoms. Only an increase in abdominal volume was the key, and ultrasound was the diagnostic tool used to confirm the pregnancy. A similar finding was observed in previous pregnancies in Cameroon with an average age of diagnosis of 15.8 ± 4.02 weeks (2). Indeed, in this population, ultrasound seems to be the only clear diagnostic tool compared with the β-HCG assay, whose level rises with a decrease in renal function (8).

We have achieved a successful pregnancy outcome up to 36 weeks and 4 days. The stabilization and maintenance of an optimal level of blood pressure control and increase in HD frequency and length of dialysis sessions were the factors that permitted us to prolong gestation and resulted in higher birth weight compared with previous reports cases. Most studies suggest that increasing dialysis time to more than 20 h per week during pregnancy results in a longer gestational period, a higher number of viable pregnancies, and a higher birth weight (5, 9). Indeed, this increase in dialysis time induces the reduction of plasmatic urea and uremic toxins, which favor a better maternal diet and better blood pressure control with good control of intra-vascular and extra-vascular fluid mass.

The increase of hemodialysis frequency and length and success to maintain the pre-dialysis blood urea between 50 and 55 mg/dl permitted us to avoid polyhydramnios. The pathophysiology of excessive amniotic fluid production is unclear, with one of the suggested mechanisms being fetal solute diuresis, due to high concentrations of urea and other uremic toxins in the maternal blood. Many studies confirmed that the incidence of polyhydramnios decreases with the increase in total dialysis time and kept blood urea at a low level (7).

The number of antihypertensive drugs was increased through pregnancy to control blood pressure. In literature, high blood pressure has been reported in about 80% of patients, with 40% developing severe hypertension, which needs three or more drugs for control (3). The mechanism of hypertension, in this case, is probably multifactorial. It can be the result of pregnancy itself or the use of high-dose EPO.

During the follow-up, the patient received two pints of packed red blood cells despite the use of EPO. This same picture was noted by Giatras et al. who observed a drop in hematocrit even during human recombinant EPO-treated pregnancies and the persistence of the requirement for blood transfusion (7).

The pregnancy was ended by cesarean section because of multiple fetal risks by vaginal delivery (maternal–fetal virus transmission, tight nuchal cords, and intrauterine growth retardation).

## Conclusion

This case adds to the growing body of evidence supporting improved pregnancy outcomes in Sub-Saharan African women undergoing hemodialysis, and it gives Cameroonian women of childbearing age on hemodialysis hope that they may be able to consider a successful pregnancy. The importance of contraception for women on HD, even if they have amenorrhea, is also highlighted in this report. Finally, this report emphasizes that the successful outcome of pregnancy in women undergoing hemodialysis in Sub-Saharan Africa is dependent on the patient’s ability to afford the high costs associated with this management, particularly in a setting without health coverage.

## Data availability statement

The raw data supporting the conclusions of this article will be made available by the authors, without undue reservation.

## Ethics statement

Ethical review and approval was not required for the study on human participants in accordance with the local legislation and institutional requirements. The patients/participants provided their written informed consent to participate in this study. Written informed consent was obtained from the patient for the publication of details of her medical case.

## Author contributions

AM, EB, and SL performed the follow-up of the patient. FS and KK did the follow-up of the child. AM, KK, SL, and GA wrote the manuscript. All the authors read and approved the final version for publication.
